# Mass Spectrometry-Based Proteomics to Unveil the Non-coding RNA World

**DOI:** 10.3389/fmolb.2018.00090

**Published:** 2018-11-08

**Authors:** Roberto Giambruno, Marija Mihailovich, Tiziana Bonaldi

**Affiliations:** Department of Experimental Oncology, IEO, European Institute of Oncology IRCCS, Milan, Italy

**Keywords:** ncRNA, mass spectrometry, SILAC, RNA-affinity purifications, miRNA, gene expression, proteome

## Abstract

The interaction between non-coding RNAs (ncRNAs) and proteins is crucial for the stability, localization and function of the different classes of ncRNAs. Although ncRNAs, when embedded in various ribonucleoprotein (RNP) complexes, control the fundamental processes of gene expression, their biological functions and mechanisms of action are still largely unexplored. Mass Spectrometry (MS)-based proteomics has emerged as powerful tool to study the ncRNA world: on the one hand, by identifying the proteins interacting with distinct ncRNAs; on the other hand, by measuring the impact of ncRNAs on global protein levels. Here, we will first provide a concise overview on the basic principles of MS-based proteomics for systematic protein identification and quantification; then, we will recapitulate the main approaches that have been implemented for the screening of ncRNA interactors and the dissection of ncRNA-protein complex composition. Finally, we will describe examples of various proteomics strategies developed to characterize the effect of ncRNAs on gene expression, with a focus on the systematic identification of microRNA (miRNA) targets.

## Introduction

Non-coding RNAs (ncRNAs) are generally defined as transcribed, but not translated RNAs. With the exception of ribosomal RNAs (rRNAs) and transfer RNAs (tRNAs)-whose role and function have long been known- the majority of ncRNAs were considered as mere transcriptional noise until their role as key-modulators of gene expression began to be unraveled. At present, it is generally accepted that ncRNAs are central players in many biological processes, such as cell proliferation, apoptosis, differentiation and development (Beermann et al., 2016; Pasut et al., 2016; Su et al., 2016). The in-depth analysis of the mammalian transcriptome by High Throughput Sequencing (HTS) technologies revealed the existence of different types of ncRNAs including: tRNAs, rRNAs, small nucleolar RNAs (snoRNAs), microRNAs (miRNAs), long non-coding RNAs (lncRNAs), circular RNAs (circRNAs), pseudogenes, and piwiRNAs. A detailed classification of ncRNAs was provided by P.P. Pandolfi and colleagues in Pasut et al. (2016).

**Figure 1:**
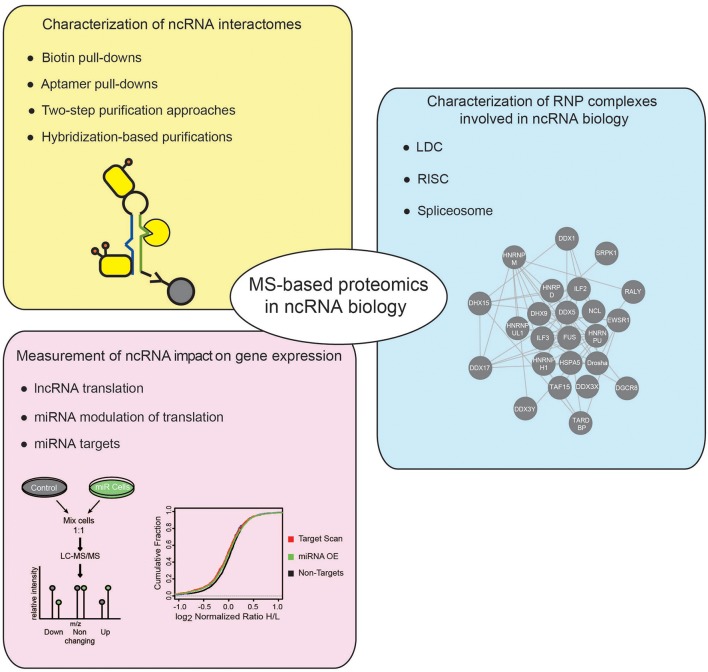
Applications of MS-based proteomics to investigate ncRNADs. Different applications of MS-based proteomics adopted to investigate ncRNAs, comprise: approaches to characterize the proteins associated to ncRNAs, using either *in vitro* or *in vivo* strategies (yellow box); MS-proteomics methods to dissect the composition of RNP complexes involved in different cellular processes regulating ncRNAs (blue box); quantitative proteomics experiments to assess the impact of ncRNAs on gene expression (pink box).

Besides HTS technologies, MS-based proteomics has emerged as powerful tool to study the ncRNA world. In this review, we will first offer a concise introduction on MS-based proteomics, with emphasis on the strategies developed for protein quantitation by MS; then, focusing on studies in eukaryotic systems, we will explain how this analytical tool has been employed to address various questions in the ncRNA research field, discussing in particular the following aspects: (i) the identification and characterization of ncRNA interactomes; (ii) the dissection of protein complexes involved in ncRNA biogenesis and function; and (iii) the measurement of the impact of ncRNAs on global gene expression (Figure 1).

### Basic principles of MS-based proteomics

Currently, the most common analytical strategy for large-scale protein identification in complex biological samples is shotgun MS-proteomics, whereby the identification of proteins from complex mixtures is carried out by tandem MS (MS/MS or MS_2_), coupled to high performance liquid-chromatography (LC). Shotgun MS-proteomics typically is carried out using a “bottom-up” MS-approach, whereby a complex protein mixture, e.g., a whole cell extract, is first digested into peptides by a specific protease. The most frequently used enzyme is Trypsin, thanks to its selectivity in cleaving the C-terminal peptide bond of lysines and arginines and its efficiency both in-solution and in-gel conditions (Vandermarliere et al., 2013). Protease-digested peptides are then separated based on their hydrophobicity by Reversed-Phase nano-Liquid Chromatography (RP-nLC) and while eluting along a gradient of increasing concentrations of an organic buffer (typically acetonitrile, ACN) are ionized and converted into gas-phase by an Electrospray Ionization (ESI) source (Fenn et al., 1989). The volatilized peptide-ions are then accelerated through an electric and magnetic field and directly transferred into the analyzer, the core of the mass spectrometer, where peptide ions are stored and then separated based on their mass-to-charge (m/z) ratios (MS1). Hybrid mass spectrometers have been designed to combine more than one analyzer, permitting not only the measurement of the exact mass-to-charge of intact peptide ions (MS1), but to also their isolation and subsequent fragmentation to generate MS/MS (MS2) fragmentation spectra. In particular, MS2 spectra contain all the mass-to-charge values of the products of peptide fragmentation which provide information to extrapolate the primary sequence of peptides (peptide sequencing). The most common peptide fragmentation techniques in MS are Collision-Induced Dissociation (CID), Higher-energy Collisional Dissociation (HCD) (Olsen et al., 2007) and Electron Transfer Dissociation (ETD) (Syka et al., 2004; Brodbelt, 2016).

Several search algorithms, typically defined as search engines, have been developed to reconstruct peptide sequences starting from (MS2); the most common are: SEQUEST (Eng et al., 1994; Yates et al., 1995), MASCOT (Perkins et al., 1999) and, more recently, Andromeda within the MaxQuant algorithm (Cox and Mann, 2008; Cox et al., 2011). All these search engines score the experimental fragmentation spectrum against the theoretical MS/MS spectrum for every peptide generated through an *in silico* digestion of the inspected proteome database with a selected protease (Paulo, 2013; Verheggen et al., 2017a). The list of candidate peptides is then filtered using a set of user-defined criteria that include the mass tolerance, the proteolytic enzyme specificity and the presence of fixed and variable modifications. The search returns a score that expresses the level of similarity between the experimental and theoretical spectra and that is therefore used as the primary parameter to discriminate correct from incorrect ID assignments. Only the best-scoring peptide matches are taken into account for the following step of protein ID and quantification. In order to convert the score into a probability-based approach several methods have been developed, among which the target-decoy searching is currently the most common one (Elias and Gygi, 2007, 2010). Specifically, a second database (the decoy) -in which all sequences are reversed and concatenated with the original one (the target)- is used to estimate the false discovery rate (FDR) of the search, assuming that both decoy matches and false positives from the target database follow the same distribution. A defined FDR threshold is then used to filter the data and remove false positive peptide identifications, up to a fixed point (typically 1%; Bantscheff et al., 2007).

MS-based proteomics is not intrinsically quantitative, first, because the ion intensity of each peptide depends not only on its amount but also on its chemo-physical properties, directly linked to their amino acid composition; second, various external factors, such as the temperature, the presence of cross-contaminants, and the quality and stability of the nano-LC system can affect the acquisition of individual peptides within a spectrum. To overcome this limitation, two main strategies have been established to extrapolate quantitative information from MS-proteomic analyses: label free quantification (LFQ) and stable isotope-labeling approaches, summarized in Table 1 and for which a detailed review has been recently published (Lindemann et al., 2017).

**Table 1:** Quantitative Mass-spectrometry-based proteomics strategies to study the ncRNA biology.

**MS-strategies**	**Strengths**	**Weaknesses**	**Applications**
Label free quantification 1) XIC 2) Spectral counting	Applicability to virtually infinite number of conditions and to any biological samples Ease of use and inexpensiveness	Requirement of high technical and experimental reproducibility Lower accuracy in protein quantification compared to isotope-based approaches	Identification of ncRNA-protein interactions Characterization of ncRNA-protein complexes
Chemical labeling 1)ICAT2) iTRAQ 3) TMT	Accuracy in protein quantification based on isotope labeling Possibility of multiplexing	Expensiveness Variable efficiency in labeling Lower accuracy in protein quantification compared to metabolic labeling	Identification of ncRNA-protein interactions Characterization of ncRNA- protein complexes Impact of ncRNA on gene expression
Metabolic labeling 1) SILAC	Accuracy in protein quantification based on isotope labeling Applicability to *in vivo* studies Minimization of quantitation error due to sample preparation Compatibility with complex purification procedures	Limited possibility of multiplexing Expensiveness in large-scale studies	Identification of ncRNA-protein interactions Characterization of ncRNA- protein complexes Impact of ncRNA on gene expression

LFQ strategies consist in the quantification of proteins using either intensity-based or spectral counting approaches. Intensity-based approaches use the extracted ion chromatogram (XIC) corresponding to the m/z ratios of each peptide. Under the assumption that the XIC value linearly correlates with the peptide abundance, this value can be employed to compare the quantity of the same peptide in different samples (Higgs et al., 2013). These approaches require high reproducibility in chromatography and rely on specific software that perform the chromatographic retention time (RT) re-alignment and the normalization of each peptide-intensity over the global chromatogram intensity (also defined as Total Ion Count, TIC). Spectral counting strategies, instead, measure the number of MS/MS spectra associated to each protein, which is assumed to linearly correlate with the protein abundance. The comparison of the number of spectra for each protein within set of experiments provide a relative index of its abundance in multiple samples. However, since the chemo-physical properties of each peptide can affect this linear correlation, accurate quantification is achieved only for proteins identified with a high number of spectra, while the quantification of low abundant and small proteins is less accurate (Bantscheff et al., 2007, 2012).

LFQ strategies are applicable to the quantification of a virtually-infinite number of samples, from any type of sources (cells, tissues, whole organisms). Nevertheless, these approaches require very high technical and experimental reproducibility and numerous biological replicates to reach confident protein quantification. LFQ approaches are reviewed in Bantscheff et al. (2012), Lai et al. (2013), Megger et al. (2013), and Lindemann et al. (2017).

In isotope-labeling strategies, proteins or peptides are labeled with stable (heavy) isotopes of various elements that make them chemo-physical identical to their natural (light) counterparts, except for a specific difference in their nominal mass. Therefore, when heavy and light proteomes are mixed, each peptide is detected in the mass spectrum as a peptide-pair, whereby the two peaks are virtually identical- except for a specific mass difference (delta mass) distinguishing them- and the ratio of the intensities of the heavy and light peptides is directly proportional to the respective abundances in the samples of origin. Hence, a relative quantification is extrapolated within the same spectrum, circumventing run-to-run variability.

**Figure 2:**
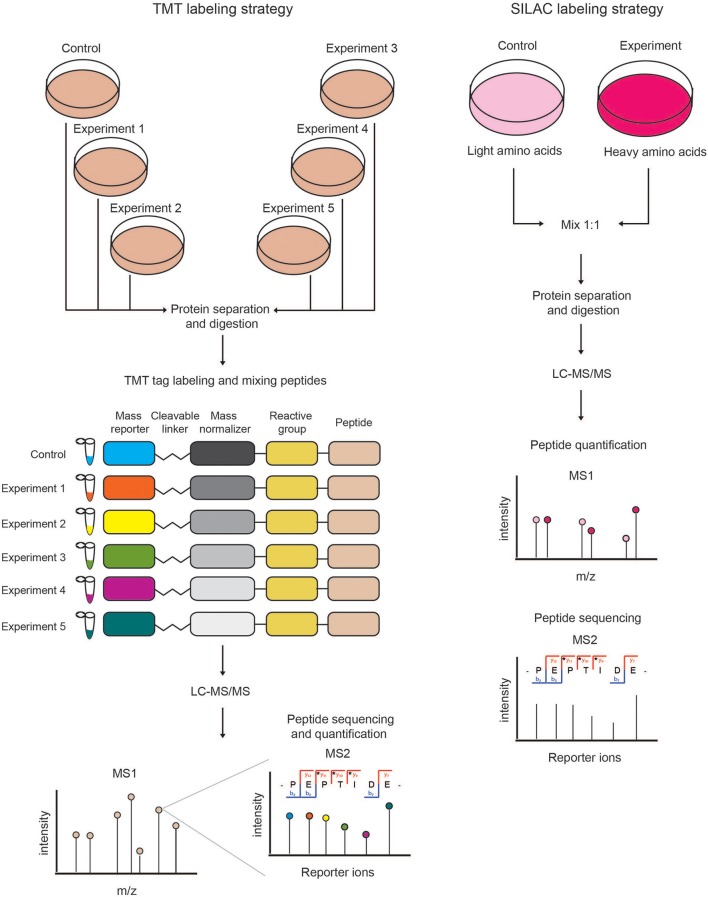
Schematic view of chemical (TMT) and metabolic (SILAC) quantitative proteomics strategies. **(Left)** In the TMT labeling strategy, protein extracts derived from different samples are reduced, alkylated, Trypsin-digested and then *in vitro* labeled using the isobaric TMT tags. The resulting peptides are mixed in equal amounts and analyzed by LC-MS/MS. At the MS1 level, the isobaric peptides appear as a single precursor ion, while at the MS2 level the different reporter ions are separated according to their mass. In this approach, peptides are identified and quantified at the MS2 level. **(Right)** In SILAC labeling strategy, cells are metabolically labeled by growing them in medium containing either light or heavy amino acids. Cells from the two experimental conditions are harvested, mixed in equal amounts and lysed to obtain a protein extract that is then reduced, alkylated and Trypsin-digested. The resulting peptides are analyzed by LC-MS/MS. Each protein-derived peptide appears as a peak-pair at the MS1 level, whereby the heavy and light peptides will be distinguishable according to their nominal mass. In metabolic labeling approaches, peptides are quantified and identified at the MS1 and MS2 levels, respectively.

Labeling of peptides with stable isotopes of C, N, H elements can be achieved *in vitro*, using a tag added covalently to the reactive side chains of amino acids, through a chemical reaction, either before or after the proteolytic cleavage. This is suitable for profiling biological samples not amenable to *in vivo* labeling, or when multiple (>3) samples must be compared. Most common methods for chemical labeling are: *Isotope-Coded Affinity Tagging* (ICAT) (Gygi et al., 1999), *Isobaric Tags for Relative and Absolute Quantitation* (iTRAQ) (Ross et al., 2004), and *Tandem Mass Tag* (TMT) (Thompson et al., 2003). ICAT was the first chemical labeling method adopted for quantitative proteomics analyses and was specifically designed for tagging cysteines with an isotopic linker region bearing a defined delta mass and a biotin module for peptide-affinity enrichment. The main limitation of ICAT lies in the fact that only cysteine-containing proteins are labeled and quantified, which reduces significantly the number of peptides profiled, and thus the depth and accuracy of proteome quantification. In iTRAQ, instead, the peptide N-terminus and the amino-groups of the lysine side chains are labeled with an isobaric tag that allows the quantification upon fragmentation in MS/MS spectra. The iTRAQ tag includes an amino-reactive group, a balance group, required to maintain constant the mass between the different isotopes, and a reporter group, used for the relative quantification at the MS2 level. The advantage of iTRAQ is the possibility of multiplexing, using up to 8 distinct isobaric tags. However, the labeling efficiency can be variable and dependent on the sample complexity, thus generating possible quantification variability. Moreover, an additional source of error can derive from the later step of labeling within the sample preparation workflow prior to MS. The TMT approach is similar to iTRAQ and consists in the isobaric-labeling of the N-terminus and lysine residues of peptides through a tag composed of four regions: a mass reporter region, a cleavable linker, a region that works as “normalizator of total mass” and a protein-reactive group (Figure 2). Pairs of TMT-labeled peptides have identical reagent structure with the same overall mass, but contain a different combination of carbon (C) and nitrogen (N) isotopes, which make them distinguishable and quantifiable upon MS/MS fragmentation. The major advantage of TMT labeling is the high multiplexing possibility, with up to 11 different samples that can be combined and profiled in parallel with no effects on the quantity and quality of the detected peptides (Thompson et al., 2003; Stepanova et al., 2018).

In the metabolic labeling strategies, the isotope is provided as a metabolic precursor to dividing cells, so that it is incorporated in the proteome during cell replication and protein neo-synthesis. This strategy is advantageous when applied to *in vivo* studies and its reliability relies on the fact that the sample mixing upon differential labeling can be carried out at the very early stages of the sample preparation workflow, thus avoiding possible biases deriving from the variability in the experimental procedure. Thus, metabolic labeling approaches are particularly well-suited for accurate protein quantitation when complex, multi-step sample preparation protocols are needed. The most popular strategy is *Stable Isotope Labeling by Amino Acids in Cell Culture* (SILAC) (Ong et al., 2004; Ong and Mann, 2006; Cao et al., 2013; Lau et al., 2014), which consists in the growth of replicating cells in culturing media complemented with the isotope-encoded version of specific essential amino acids, allowing the incorporation of stable isotopes into proteins during *de novo* protein synthesis. In particular, cells are grown in an *ad hoc* medium that contains either the heavy (^2^H, ^13^C, and ^15^N) or light (H, ^12^C, and ^14^N) versions of lysine and arginine for a number of passages that enable their full incorporation into the proteomes (Figure 2). Heavy (H) and light (L) cells are then harvested, mixed in equal amounts and proteins are separated, digested and subjected to MS-analysis. In this setup, each protein-derived peptide exists in MS1 as a peak-pair, with the H and L counterparts being distinguishable by the delta mass. Hence, the intensity of the two peaks is an indicator of the corresponding amount in the two SILAC states. SILAC labeling has been applied to a wide variety of studies, including protein expression profiling, global PTM analysis, protein-protein and nucleic acids-protein interaction analyses. Moreover, a modified version of the standard SILAC, named pulsed-SILAC (pSILAC) was successfully adopted to quantitatively assess protein translation dynamics, as described below.

### MS-based proteomics for the systematic analysis of ncRNA-protein interactions

In the last two decades, various strategies have been developed to comprehensively analyze ribonucleoprotein (RNP) complexes. They can be grouped based on whether the RNAs or the proteins are used as baits for RNP complex enrichment and dissection: in the *RNA-centric* approaches, the RNA of interest is used as a bait to enrich and identify the respective protein interactors by MS- proteomics; in *protein-centric* approaches, specific RNA-binding proteins (RBPs) are used as baits to characterize the respective RNA interactors by RNA-sequencing analysis. Given the focus of this review on proteomic methods in ncRNA research, we will focus only on RNA-centric strategies.

Historically, *in vitro* RNA pull-down coupled to MS-based proteomics has been the most successful approach to identify RBPs regulating various RNAs. Improvements of this method led to the development of *in vivo* RNA immuno-purification assays, which enabled the purification of native RNPs from living cells. In the last few years, more global approaches to study RBPs have shifted the focus from the analysis of single RNAs to the comprehensive investigation of global cellular RNA-interactomes (Castello et al., 2013).

### RNA affinity-purification assays

RNA affinity-purification assays coupled to MS-based proteomics are used for the purification of ncRNA interactomes, both *in vitro* and *in vivo* [reviewed in Yang et al. (2015), Jazurek et al. (2016), Faoro and Ataide (2014)]. In this assay, a co-transcriptionally labeled, or tagged, RNA is used as a bait that can be either directly incubated with the protein extract, or bound to a solid support, prior to incubation. The proteins interacting to the labeled/tagged RNA are first immuno-precipitated and then identified by MS-proteomics. Variants of this basic RNA affinity-purification strategy have been developed, with technical improvements that have led to: (a) the implementation of different tagging molecules that favor the proper folding of the bait-RNA and minimize structural interference during the assembly of RNP complexes; (b) the introduction of two-step purification systems that enable more specific elution; (c) quantitative proteomics strategies that potentiate the discrimination of specific RNA-interactors from unspecific binders. Among them, SILAC is particularly well-suited to analyze low abundant and/or transient interactions, which is often the case when investigating protein-RNA associations.

### Biotin-labeled RNA pull-downs

Biotin (vitamin H) is the most common tag used in RNA pull-down experiments. It can either be incorporated during *in vitro* transcription, when the RNA is synthesized in the presence of biotinylated nucleotides, or it can be added at the 3′- or 5′-ends of pre-synthetized RNA by enzymatic reaction. Upon incubation with the protein extract, proteins bound to biotinylated-RNAs are purified through streptavidin beads. The interaction between biotin and streptavidin is very strong, highly specific and resistant to high salt concentrations, high temperatures and extreme pH, which is particularly advantageous because it allows performing very stringent washes (Jazurek et al., 2016). However, this interaction is so potent that elution using soluble biotin in excess is precluded and the elution is thus performed with strong denaturing buffers, or with RNase A, which may release sticky proteins from the beads. Also, the incorporation of biotinylated nucleotides can interfere with the proper folding of the bait-RNA, thus impairing the proper binding of some interactors to specific secondary structures of the RNA; on the other hand, 3′- or 5′-end tagging can be inefficient (Jazurek et al., 2016). In spite of these limitations, biotin has led to the characterization of the interactomes of various lncRNAs, such as HOTAIR, *Firre* and lincRNA-p21 (Rinn et al., 2007; Huarte et al., 2010; Hacisuleyman et al., 2014).

### Aptamers

Aptamers are short oligonucleotides or peptides that bind with high affinity to specific target molecules. They are very attractive RNA-tags, thanks to their suitability for purification under native conditions, for both *in vivo* and *in vitro* experiments. Some aptamers are naturally-occurring RNA stem-loop sequences (e.g., MS2 and PP7), while others are derived from library screenings (S1, D8, tobramycin and streptomycin). The MS2 and PP7 aptamers are based on the bacteriophage system: they bind with high specificity to the *Escherichia coli* bacteriophage coat protein MS2 (MS2cp) and *Pseudomonas aeruginosa* bacteriophage coat protein PP7, respectively (Johansson et al., 1997; Lim et al., 2001). In a RNA pull-down setting, the bait RNA is tagged at the 3′- or 5′- end with repeats of either the MS2-binding or the PP7-binding RNA stem-loops, which enable the affinity-purification of the RNPs containing the tagged RNA by using immobilized MS2 or PP7, respectively. MS2 can also be fused to the maltose-binding protein (MBP), which allows the selective elution of the RNP complexes by the addition of molar excess of soluble maltose (Zhou and Reed, 2003). This purification strategy, in combination with SILAC-based quantitative protein profiling, was applied to study the proteins associated to the lncRNA HOTAIR (Meredith et al., 2016). MS2-tagged HOTAIR and control-RNA were incubated with nuclear extracts from differentially SILAC-labeled HeLa and MDA-MB-231 cells; binding proteins were purified via MS2-MBP conjugated to amylose resin and *bona-fide* HOTAIR interactors were distinguished from the background proteins on the basis of their bait-over-control SILAC ratio. With the same approach, the interactomes of the lncRNAs MEG3 and treRNA were also characterized, upon the implementation of an additional cross-linking step, by either UV or formaldehyde, to stabilize the RNP complexes (Gumireddy et al., 2013; Liu et al., 2015).

### Two-step purification approaches

Aptamers are also commonly used in the two-step purification strategies, developed to increase the purity of the isolated RNP complexes reducing the contamination from nonspecific proteins. The first two-step purification method, named RAT (*RNA Affinity in Tandem*), was developed by Hogg and colleagues for the characterization of proteins associated to the 7SK RNA (Hogg and Collins, 2007). In the RAT approach, the 7SK RNA is tagged with both PP7 and tobramycin aptamers and expressed in cells together with the recombinant PP7 carrying a TEV protease cleavage site. PP7 is used for the first purification step of native RNPs; upon elution with TEV, the tobramycin resin is used for the second purification step. RAT paved the way to various two-step purification variants, the majority taking advantage of the MS2 aptamer. For instance, Slobodin and Gerst developed the RaPID (*RBP Purification and Identification*) method that employs a MS2-based fluorescent reporter (MS2-CP-GFP) fused with the streptavidin-binding protein (SBP) tag, thus allowing both the visualization of the mRNAs bearing the MS2 aptamer and the purification of the protein interactors of the MS2-tagged RNAs by streptavidin beads (Slobodin and Gerst, 2010). Tsai and colleagues developed the MS2-BioTRAP [*MS2 in vivo Biotin Tagged RNA Affinity Purification*, (Tsai et al., 2011)], where the cells are co-transfected with both the MS2-tagged RNA of interest and the MS2 protein fused with the HB-tag, which, in turn, consists of the following elements: a hexahistidin tag, a TEV cleavage site and a bacterially-derived signal peptide for *in vivo* biotinylation in mammalian cells (Tagwerker et al., 2006). Upon expression, the MS2-tagged RNA with its interacting proteins is specifically bound to the MS2-HB biotinylated protein and the whole MS2-RNP complex is first stabilized by UV cross-linking, and then purified on streptavidin beads. Moreover, Tsai and colleagues used SILAC labeling to distinguish genuine interactors from unspecific background. The Gorospe lab improved further this method developing the MS2-TRAP (*MS2-Tagged RNA Affinity Purification*), whereby the MS2-tagged RNAs and the chimeric MS2-glutathione-S-transferase (MS2-GST) protein are co-expressed in mammalian cells, thus permitting the purification of native RNP complexes formed on the MS2-tagged RNA with a glutathione-sepharose resin (Yoon et al., 2012).

### Hybridization-based purification strategies

Hybridization-based purification strategies have been originally used for the systematic mapping of lncRNAs along the genome; subsequently, these techniques have been applied also to the investigation of lncRNA interactomes. For example, Chu and colleagues modified the ChIRP-Seq technique (Chu et al., 2011) into the ChIRP-MS approach (*Comprehensive Identification of RNA-binding Proteins by Mass Spectrometry*), to enable the large-scale identification of lncRNA-bound proteins *in vivo*. The workflow of both methods is the same: cells are first cross-linked *in vivo*, chromatin is extracted and sheared by sonication, and then biotinylated oligonucleotides complementary to the complete lncRNA sequence are added and let hybridize with the lncRNA. The hybrids, which comprise the target lncRNA, the associated cross-linked proteins and the chromatin, are purified using streptavidin beads. While in ChIRP-Seq the aim is to capture and sequence the genomic DNA binding to the lncRNA of interest, in ChIRP-MS the biotin-elution is followed by de-cross-linking, protein extraction and MS-analysis. This method was successfully applied to comprehensively identifying the Xist- interactome (Chu et al., 2015). A very similar method is CHART-MS (*Capture Hybridization Analysis of RNA Targets by Mass Spectrometry*), a derivate of CHART-Seq (Simon et al., 2011) that differs from ChIRP mainly by the smaller size of the biotinylated probes used for purification, which are exclusively complementary to the lncRNA domain binding to DNA. CHART-MS was successfully used to identify the proteins interactors of the lncRNAs MALAT1 and NEAT1 (West et al., 2014).

The Xist interactors were also studied by the RAP-MS (*RNA Antisense Purification by Mass Spectrometry*) method (McHugh et al., 2015) that is a proteomic adaptation of the RAP method (Engreitz et al., 2013). Although also RAP and RAP-MS make use of biotinylated antisense probes, they are more efficient in RBPs identification than previous strategies, thanks to a number of improvements, which include: (a) the use of longer (>60 nucleotides) biotinylated antisense probes that enable the formation of very stable RNA-DNA hybrids, thus permitting more stringent washing steps during the lncRNA–protein complex purification; (b) the use of UV instead of formaldehyde for cross-linking, which allows fixing exclusively the direct binders to the RNAs; (c) SILAC-based protein profiling was employed in combination with RAP, to facilitate the discrimination of specific binders from background proteins. All these aspects have made RAP the elective strategy for the combined identification of the DNA-binding sites and protein-interactors of lncRNAs, such as in the case of the Firre lncRNA (Hacisuleyman et al., 2014).

Another attractive hybridization-based method that enables to directly monitor local RNPs *in vivo*, is PAIR (*PNA (Peptide Nucleic Acid)-Assisted Identification of RBPs*), which was successfully applied to study proteins associated to *ank*, a pan-neuronal dendritic mRNA (Zielinski et al., 2006). The PAIR method is based on the use of specific RNA-binding probes (PNAs) that hybridize with high specificity and selectivity to the endogenous target RNA and are cell permeable, thanks to the presence of a cell-penetrating peptide (Margus et al., 2012). PNAs contain also a photo-activated compound, which covalently cross-links to the associated RBPs when cells are exposed to UV light and thus stabilizes all direct interactors, allowing stringent washing steps. RNP complexes are then purified through biotinylated sense (antisense to PNA) oligonucleotides, coupled to streptavidin beads; afterwards co-associated proteins are identified by MS-based proteomics.

### Pitfalls in ncRNA-interactor identification by MS-based proteomics

The choice of the ideal RNA affinity-purification strategy for specific research goals should take into account the various experimental parameters that may influence the isolation of RNP complexes prior to proteomics analysis, which include: (1) the expression level and folding of the RNA used as bait; (2) the cellular localization of the RNA; (3) the type of cell-lysis protocol employed; (4) the RNA-to-RBP stoichiometry; and (5) the stability of the RNP complex under investigation. These experimental issues are reviewed in more detail in Oeffinger (2012).

*In vitro* RNA affinity-purification approaches are generally the first experimental choice because they are relatively fast and manageable and require overall small amount of starting material. However, *in vitro* purifications usually suffer from a large excess of bait-RNA that affects the physiological RNA-to-RBP stoichiometry. In addition, *in vitro* synthesized RNA might not fold properly and the protein extract used for the purification is not restricted to the cellular compartment where the endogenous RNA resides. All together, these aspects might favor the formation of artificial and unspecific protein-RNA interactions (McHugh et al., 2014).

*In vivo* RNA affinity-purification strategies are generally more challenging from a technical point of view, but they allow preserving the native protein-RNA interactions, catching the physiological RNP complexes in the cell. Such experiments have been mainly carried out using highly abundant or over-expressed RNAs, as baits. In the latter condition, however, the over-expressed RNA may display either aberrant cellular localization or altered RNA-RBP stoichiometry (Riley et al., 2012; Jazurek et al., 2016).

In addition, both *in vitro* or *in vivo* RNA affinity-purification strategies are often biased toward the most abundant RBPs, such as hnRNPs, RNA helicases, ribosomal and spliceosomal proteins, which might promiscuously associate with any RNA sequence (Butter et al., 2009; McHugh et al., 2014). To this regard, crosslinking strategies which stabilize protein-RNA interactions and thus allow using stringent washing conditions are useful both to limit the cross-contamination from abundant sticky proteins and to increase the identification of transient and weak, although specific, interactions. In general, crosslinking strategies are particularly suitable for the identification of the protein interactors of low abundant RNAs (McHugh et al., 2014; Ferrè et al., 2016).

RNA affinity-purifications in non-denaturing conditions followed by MS-based proteomic analysis often leads to the identification of hundreds of proteins, due to general stickiness of RNP complexes under investigation and to the high sensitivity of modern mass spectrometers. In this context, quantitative MS approaches, such as SILAC, iTRAQ, TMT are crucial to discriminate “true” interactors from the protein background (Meyer and Selbach, 2015; Aebersold and Mann, 2016). Nevertheless, also quantitative MS-approaches are affected by the over-representation of highly abundant proteins in the list of the identified specific interactors (Duncan et al., 2010; Hu et al., 2016; Ankney et al., 2018).

### MS-based proteomics for the characterization of multi-protein complexes involved in ncRNA processing and function

Various RNA-centric methods have been adopted for the characterization of RBPs involved in miRNA biogenesis. For instance, a method exploiting the endoribonuclease Csy4, a part of the CRISPR system in *Pseudomonas aeruginosa*, was applied for the purification of RBPs associated to pre-let-7a, pre-miR-200a and pre-miR-342 (Lee et al., 2013). This purification method relies on various Csy4-specific features: (i) the selective recognition and cleavage of a 16-nt hairpin sequence; (ii) the strong affinity for the substrate; (iii) the presence of a histidine in the catalytic domain, which -when mutated to alanine H29A- does not affect the substrate binding affinity and specificity; *iv)* the possibility of rescuing the cleavage activity of Csy4 H29A by imidazole. The peculiar conditional activity of Csy4 H29A, together with the high selectivity and affinity of the enzyme for its substrate, permits a very selective affinity-purification of RNP complexes. Typically, pre-miRNAs are tagged with 16-nt hairpin sequences at their 5′-end. Upon incubation with the cellular extract, the complex comprising the Csy4 hairpin tagged-pre-miRNA and the co-associated proteins is captured by biotinylated-Csy4 H29A, immobilized on avidin resin; upon stringent washing steps, the RNPs are eluted by imidazole. The selective transcript isolation with imidazole ensures very low background contamination which allows omitting a SDS-page purification step prior to MS, thus favoring the detection of low-abundant RBPs.

A RNA-centric approach was also employed by the group of G. Meister to annotate the most comprehensive dataset of RBPs involved in miRNA biogenesis: they used 72 *in vitro* synthetized pre-mRNA bearing the same 5′ extension, complementary to biotinylated 2′-O-methyloligonucleotides, to uncover 180 proteins that show preferential or selective binding to single, or small sets of precursor-miRNAs (Treiber et al., 2017). The results collected in this study corroborated the model whereby miRNA biogenesis is a dynamic process mediated by a temporally-defined association of different RBPs.

In addition to RNA-centric approaches, classical strategies for multi-protein complexes purification, typically based on the overexpression of the protein of interest, followed by immuno-purification and MS-identification of the co-associated proteins, have been employed to characterize biochemically the nucleoprotein machineries involved in miRNA biogenesis and function. In 2004, three groups independently identified the Microprocessor complex, composed of DROSHA and DGCR8 and responsible for the pri-to-pre- miRNA cleavage step (Denli et al., 2004; Han et al., 2004; Liu et al., 2004). Immuno-precipitation of a FLAG-tagged version of both DROSHA and DGCR8 led to the identification of 21 additional auxiliary proteins that associate to the Microprocessor, forming a multi-protein complex named *Large Drosha Complex* (LDC). These auxiliary proteins are required to modulate the ribonuclease activity of the core dimer (Liu et al., 2004; Shiohama et al., 2007). The RISC (*RNA-Induced Silencing Complex*) was characterized with a similar strategy, in the same period (Chendrimada et al., 2005; Haase et al., 2005; Höck et al., 2007).

### MS-based quantitative proteomics to study the function of ncRNAs

#### Proteomics analyses to assess the translation of ncRNA into polypeptides

Advances in sequencing technologies have led to the discovery that pervasive transcription in eukaryotes produces an excess of lncRNAs (Djebali et al., 2012). Initially these molecules were believed not entailing a protein-coding potential, but subsequent ribosome profiling analyses have demonstrated that lncRNAs can interact with the translational machinery (Bazzini et al., 2014; Ingolia et al., 2014; Calviello et al., 2016). Nevertheless, the question whether the observed ribosome-footprints on lncRNAs and the presence of small open reading frames (smORFs) may truly reflect an active translation into short polypeptides is still debated (de Andres-Pablo et al., 2017; Verheggen et al., 2017b). Several groups demonstrated- first in fruit-fly and zebrafish and then in mammalian cells- that some very short polypeptides (micropeptides) are produced from putative lncRNA transcripts and are functionally relevant (Pauli et al., 2014; Albuquerque et al., 2015; Anderson et al., 2015; Nelson et al., 2016). While in all these studies micropeptides were discovered starting from RNA-seq analyses followed by *in silico* prediction, the Pandolfi group took advantage of MS-based proteomics to identify for the first time a smORF encoded by the lncRNA LINC00961, thus demonstrating its translatability (Matsumoto et al., 2017). Nonetheless, as of today, < 1% of the micropeptides encoded by lncRNAs has been experimentally validated by MS-proteomics (Volders et al., 2015), raising concerns about the technical possibility to annotate micropeptides by MS. Recently, Verheggen and colleagues have systematically addressed the possible biases in micropeptide detection by MS, assessing how various factors, such as size, amino acid composition, abundance and half-life- could affect the identification of peptides encoded by lncRNAs (Verheggen et al., 2017b). They observed that MS is not specifically biased against the detection of lncRNA-encoded micropeptides, so that their lack of detection with this technique may indeed be suggestive of their overall absence. Recently, U. Ohler and colleagues developed the RiboTaper statistical approach to identify translated regions from ribosome profiling data (Calviello et al., 2016). Interestingly, the experimental proteome which they acquired by MS-proteomics showed excellent match with the putative proteome predicted by RiboTaper. Indeed, only 504 ORFs within noncoding genes were identified as translated, with the majority belonging to pseudogenes; this is in line with the idea that most lncRNAs are, in fact, non-coding.

#### Quantitative proteomics to characterize miRNA function and targets

In the last decade, miRNAs have emerged as pervasive post-transcriptional regulators, with each miRNA being able to dampen the expression of hundreds of targets, simultaneously. They repress target genes by either destabilizing mRNAs and/or inhibiting their translation. MiRNAs bind their targets through the interaction of a seed region with the matching binding site located in the 3′ untranslated region (3′ UTR) of the transcript. One mRNA may have different variants bearing 3′ UTRs regions of different length. Any variation in the 3′ UTR length of a transcript can affect its regulation and stability as a consequence of the changes in the repertoire of regulatory elements present within the region, including regulatory RBPs and binding sites for miRNAs (Mihailovic et al., 2012; Tian and Manley, 2013). Although many computational approaches for miRNA targets prediction have been developed, *in silico* predictions overall lack accuracy, as they do not take into account the 3′ UTRs heterogeneity, as well as imperfect mRNA/miRNAs match. Hence, experimental validation of miRNA target prediction is mandatory when investigating the genuine biological activity of individual miRNAs in a specific context. Vinther and his colleagues pioneered the use of MS-based proteomics for the unbiased experimental identification of miRNA targets using SILAC-based quantitative profiling of protein level changes, upon miR-1 overexpression (Vinther et al., 2006). Although only 12 high-confident miR-1 targets were identified, this study represented the proof-of-concept that a single miRNA can reduce the translation of several proteins in parallel, and showed that SILAC-based quantitation is particularly suitable for assessing the very mild protein changes induced by miRNA modulation. The steady-state proteomic analysis may, however, underestimate the effect of differential turnover among proteins, as well as the combinatorial effect of multiple regulatory events impinging on the overall protein levels. For example, on the one hand, very stable proteins may result unaltered when the proteomics analysis is performed too shortly after miRNA modulation; on the other hand, if MS analysis is carried out too late, the proteome changes may be the result of both direct (miRNA activity) and indirect (e.g., the modulation of some transcription factors) events.

**Figure 3:**
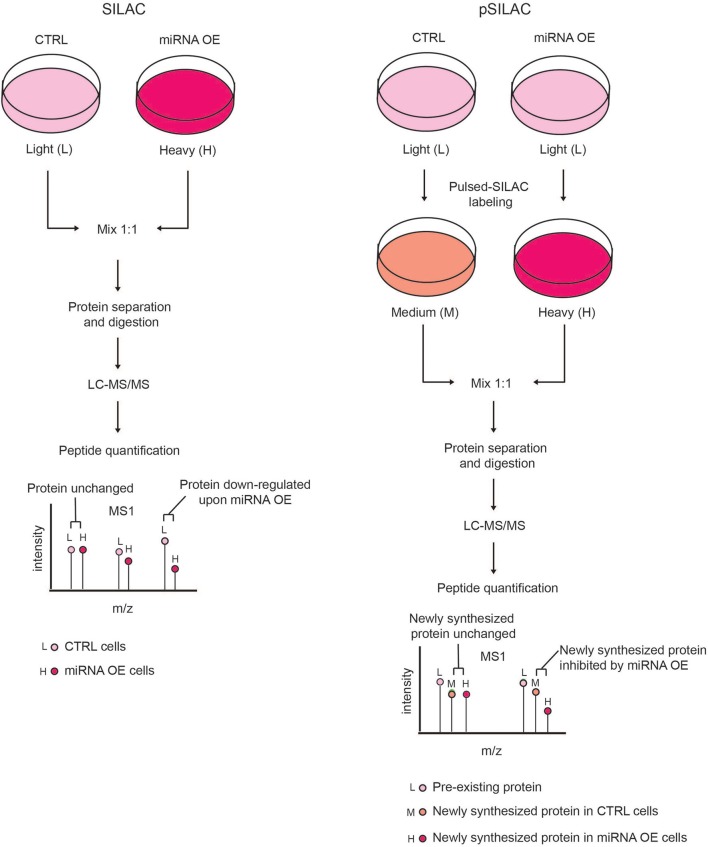
Comparison of SILAC and pSILAC strategies to assess miRNA impact on protein levels. **(Left)** In the SILAC labeling strategy, cells are grown for several doublings in SILAC medium containing either light (L) or heavy (H) amino acids and then subjected to different treatments, e.g. transfected with either control (CTRL) or miRNA over-expressing (OE) vectors. Cells from both conditions are then harvested, mixed in equal ratio and analyzed by LC-MS/MS as a single sample. Each protein-derived peptide will appear as a peak-pair that reflects a mixture of both the pre-existing and newly synthesized proteins. The intensity ratio of the two peaks within each peak-pair is proportional to the amount of the original peptide in the CTRL and miRNA OE cells, allowing the quantification of proteins in the two conditions. A peptide ratio (H)/(L) ≅ to 1 indicates that the protein level is unchanged, while a peptide ratio (H)/(L) < 1 indicates that the protein level is reduced by miRNA OE, hence the protein is a putative miRNA target. **(Right)** In pSILAC, CTRL and miRNA OE cells are initially grown in SILAC media, containing light amino acids. Subsequently, both CTRL and miRNA OE cells are pulsed with distinct SILAC media containing either medium-heavy (M) or heavy (H) amino acids. After a short time interval, cells are harvested, mixed in 1:1 ratio and the extracted proteins are digested and LC-MS/MS analyzed. Each protein-derived peptide will appear as a peak-triplet, whereby the pre-existing proteins appear as (L) peaks, while newly synthesized ones display either (M) or (H) peaks and the ratio of (H) over (M) peaks is the result of the miRNA OE impact on protein neo-synthesis. Newly synthesized proteins with a SILAC ratio (H)/(M) < 1 are putative miRNA targets while non-targeted proteins show a SILAC ratio (H)/(M) ≅ to 1.

This limitation can be overcome by using the pulsed-SILAC (pSILAC) strategy, which was developed by M. Selbach in 2008 and represents a technological milestone for experimental miRNA target identification by MS (Selbach et al., 2008). In pSILAC, both control and treated cells are pulse-labeled with two distinct versions of the heavy amino acids, so that pre-existing proteins are visualized in the light channel, and the newly synthesized proteins from different functional states (e.g., untreated and perturbed) exist in the “heavy” and “medium-heavy” channels, respectively. The comparison of the heavy and medium-heavy channels for the same proteins reveals the impact of a specific perturbation (e.g., miRNA modulation) only on protein neo-synthesis/translation (Figure 3). Specifically, Selbach and colleagues globally assessed the effect of protein translation upon the modulation of both endogenous and exogenous miRNAs in HeLa cells and showed that a single miRNA can affect the expression of hundreds of proteins. Pulsed-SILAC allows a better identification of direct miRNA targets, therefore it is well-suited to distinguish targets of distinct miRNAs belonging to the same family (Ebner and Selbach, 2014), which is almost impossible with *in silico* predictions.

While studies using transient modulation of miRNAs are almost exclusively focused on target identification, the studies involving the stable overexpression or down-regulation of miRNAs can offer additional insights into the physiological role of the miRNA under investigation. For example, Baek and colleagues interrogated the effect of *mir-223* gene knockout in mouse neutrophils. They isolated bone marrow hematopoietic progenitors from wild-type and mir-223 deficient mice and differentiated them *in vitro* in SILAC conditions, to analyze mir-223 targets by quantitative proteomics (Baek et al., 2008). To discern direct from indirect targets, they intersected the list of up-regulated proteins with miRNA target *in silico* prediction.

Although the experimental validation of the predicted targets through quantitative proteomics accurately reflects changes in their protein abundances, these changes might still be the sum of both the direct activity of miRNAs and other adaptive responses of the cell. We investigated this aspect in 2015, by carrying out an integrated analysis of SILAC-based proteomics, transcriptomics, *in silico* prediction and in-depth 3′ UTR-analysis, in full-blown B cell lymphoma, where we modulated the expression of the miR-17-92 cluster. This integrated analysis of multiple -omics data revealed the importance of 3′ UTR shortening in defining the effective miR-17-92 activity in full-blown B cell lymphoma, with the miRNA cluster shifting from an oncogenic to a tumor-suppressor role due to differences in mRNA landscape at different stages of tumor progression (Mihailovich et al., 2015).

In addition to SILAC and its variations, also chemical labeling methods -such as ICAT, iTRAQ and TMT- were employed in various miRNAs studies, when either the model systems under investigation were not amenable to metabolic labeling, or more extensive multiplexing was required. For instance, Li and colleagues isolated splenic B cells from three miR-146a-overexpressing transgenic mice and three controls and labeled them with 6-plex isobaric TMT tags, which enabled the simultaneous analysis of all samples in a single MS run. They identified and quantified over 5,000 proteins, from which ~200 were differentially expressed between B cells from miR-146a transgenic mice and controls (Li et al., 2017).

Taken together, MS-based quantitative proteomics has contributed significantly to the large-scale identification of miRNA targets, thanks to the implementation of *ad hoc* strategies and experimental designs.

## Conclusions and future perspectives

Different classes of ncRNAs, including miRNAs and lncRNAs, have been described as key regulators of gene expression; with the increasing knowledge gained through HTS, novel and less characterized classes of ncRNAs will be probably included in the same regulatory group, as was the case for the recently reported circRNAs (Huang et al., 2017). A feature in common to all these molecules is their association with proteins to form RNP complexes, whose composition, organization and dynamics is spatial- and temporal- specific, and dependent on both intra- and extra- cellular stimuli. Both protein-centric and RNA-centric high-throughput methods are required to characterize the dynamicity of ncRNP complexes. The development of the *in vivo RNA-Interactome Capture* (RIC) method, coupled with MS-proteomics analysis (Castello et al., 2012) enabled the systematic and comprehensive identification of RBPs from various cell types and organism models (Baltz et al., 2012; Kwon et al., 2013; Mitchell et al., 2013; Marondedze et al., 2016; Nandan et al., 2017), offering important insights into RNA biology. The RIC approach, in fact, almost doubled the number of annotated RBPs and uncovered dozens of the so-called “enigma” RBPs, for which the corresponding RNA-binding module and RNA-partners are unknown (Hentze et al., 2018). The continuous annotation of RBP compendia has paved the way to novel approaches to study RBPs; for example, Zappulo and colleagues identified a sub-population of 29 RBPs specifically localized in neurites, by comparing a local proteome acquired from neurites with a published repository of 1,542 RBPs (Gerstberger et al., 2014; Zappulo et al., 2017). Interestingly, a parallel ncRNA-analysis carried out in the same neuronal compartment unraveled 12 lncRNAs of unknown function and 41 circRNAs, thus suggesting that the newly identified neurite-specific RBPs might be involved in the regulation of these ncRNAs.

Very recently three similar strategies, named respectively OOPS, XRNAX, and PTex, have been developed as very promising global approaches to expand the current knowledge on RBPs and RNA-binding domains (Queiroz et al., 2018; Trendel et al., 2018; Urdaneta et al., 2018). They all adopt an organic phase separation step for the extraction and enrichment of UV-crosslinked RNA-protein complexes from whole cell extracts. The isolated complexes are then analyzed by either RNA-sequencing or MS-based proteomics for the identification of the RNA- and protein- constituents, respectively. By avoiding any RNA/protein affinity-purification step, these methodologies allow more systematic and unbiased characterization of RNA-protein interactions, including less characterized ncRNA species, in both eukaryotic and prokaryotic systems.

Although more extensively employed in eukaryotes, most of the approaches based on affinity purification coupled to MS-based proteomics discussed in this review can in principle be adopted to identify ncRBPs in prokaryotes, a less developed field which has been recently reviewed in Holmqvist and Vogel (2018). For example, already in 2009 Said et al. used the MS2-aptamer to identify *in vivo* the proteins bound to various small non-coding RNAs (sRNAs) from *Salmonella Typhimurium* (Said et al., 2009). Similarly, Rieder et al. employed *in vitro* MS2-tagged RNA pull-downs to characterize the RBPs of sRNAs from *Helicobacter Pilori* (Rieder et al., 2012). Differently, Osborne and colleagues developed the SSAC-MS/MS (Sequence-Specific Affinity Chromatography and tandem Mass Spectrometry) method, in which biotinylated cDNA probes, complementary to the sRNA target, were used to purify UV-crosslinked sRNA-RBP complexes formed *in vivo*. SSAC-MS/MS was employed to define the interactomes of the iron-responsive PrrF and PrrH sRNAs in *Pseudomonas aeruginosa* (Osborne et al., 2014). All these approaches are based on the characterization of the RBPs interacting with a specific tagged sRNA used as bait. Very recently, the successful application of the PTex strategy in *Salmonella Typhimurium* demonstrated the possibility of applying an unbiased and global identification of the numerous RNP complexes in living prokaryotic systems (Urdaneta et al., 2018).

With the expanding knowledge on multifunctional RBPs, the RNA field has focused its interest on understanding the link between post-transcriptional regulation and signaling pathways that respond to environmental and/or developmental stimuli. Along this line, the modulation of the protein components of RNPs by post-translational modifications (PTMs) has gained much attention. It has been suggested that various PTMs, such as phosphorylation, methylation, glycosylation, acetylation, NEDDylation and ubiquitination, modulate both protein-protein and protein-RNA interactions within RNPs, thus influencing both the processing, stability, turnover and translation of mRNAs (Will and Luhrmann, 2011; Chen and Moore, 2014; García-Mauriño et al., 2017; Gonçalves et al., 2018) and the biogenesis and function of ncRNAs (Heo and Kim, 2009; Herbert et al., 2013; Hu et al., 2014; Jee and Lai, 2014; Golden et al., 2017). Interestingly, RBPs are the most frequently arginine-methylated proteins in the mammalian cells (Liu and Dreyfuss, 1995; Blackwell and Ceman, 2012) and arginine-methylation was shown to be required for the correct assembly and function of various RNP complexes, such as the spliceosome. The spliceosome is the largest RNP complex characterized in cells so far, comprising 5 snRNAs, around 50 small-nuclear RNPs (snRNPs) and more than a hundred of non-snRNP proteins that are required for proper pre-mRNA splicing (Wahl et al., 2009; Newman and Nagai, 2010). The composition of the spliceosome is highly dynamic and regulated by several PTMs, among which arginine methylation. In particular, the core components SmD1, SmD3, and SmB are methylated by protein arginine methyltransferase 5 (PRMT5) to mediate the correct assembly of some snRNP particles within the Spliceosome, in the cytoplasm compartment [Reviewed in Yu (2011)]. While a lot has been described on the role of spliceosome-methylation, the extent and function of this modification in other RBPs are largely unknown. In 2013, through a systematic analysis of protein arginine-methylation by MS we reported for the first time that the LDC is hyper-methylated at arginine residues, suggesting a possible regulatory role of this modification in miRNA biogenesis (Bremang et al., 2013).

MS-based modification-proteomics will play a pivotal role in the future to dissect the extent and regulatory impact of arginine methylation, as well as other PTMs, in defining the biogenesis, stability, location and function of RBPs.

## Author contributions

RG and MM wrote the draft. TB proposed the topic and revised the text.

### Conflict of interest statement

The authors declare that the research was conducted in the absence of any commercial or financial relationships that could be construed as a potential conflict of interest.
